# Methods for trustworthy nutritional recommendations NutriRECS (Nutritional Recommendations and accessible Evidence summaries Composed of Systematic reviews): a protocol

**DOI:** 10.1186/s12874-018-0621-8

**Published:** 2018-12-05

**Authors:** Bradley C. Johnston, Pablo Alonso-Coello, Malgorzata M. Bala, Dena Zeraatkar, Montserrat Rabassa, Claudia Valli, Catherine Marshall, Regina El Dib, Robin W. M. Vernooij, Per O. Vandvik, Gordon H. Guyatt

**Affiliations:** 1 Department of Community Health and Epidemiology, Faculty of Medicine, Dalhousie University, Halifax, Canada; 2 Department of Health Research Methods, Evidence and Impact, McMaster University, Hamilton, ON Canada; 3 Iberoamerican Cochrane Centre Barcelona, Biomedical Research Institute San Pau (IIB Sant Pau), Barcelona, Spain; 4 CIBER de Epidemiología y Salud Pública (CIBERESP), Barcelona, Spain; 5 Chair of Epidemiology and Preventive Medicine, Department of Hygiene and Dietetics, Jagiellonian University Medical College, Krakow, Poland; 6 Cochrane Consumer and Honorary Patron of the Guidelines International Network, Wellington, New Zealand; 7 Institute of Science and Technology, Unesp - Univ Estadual Paulista, São José dos Campos, Brazil; 8 Department of Research, Netherlands Comprehensive Cancer Organisation (IKNL), Utrecht, The Netherlands; 9 Institute of Health and Society, Faculty of Medicine, University of Oslo, Oslo, Norway; 10 Department of Medicine, Innlandet Hospital Trust-division, Gjøvik, Norway; 11 Department of Medicine, McMaster University, Hamilton, ON Canada

**Keywords:** Nutrition, Guidelines, Recommendations, Evidence-based, Patient engagement

## Abstract

**Background:**

Recent systematic reviews and editorials suggest that many organizations that produce nutritional guideline recommendations do not adhere to internationally recognized standards set forth by the Institute of Medicine (IoM), Guidelines International Network (GIN), Appraisal of Guidelines Research and Evaluation (AGREE), and Grading Recommendations, Assessment, Development and Evaluation (GRADE).

**Methods:**

The potential solution is an independent group with content expertise and skilled in the methodology of systematic reviews and practice guidelines to produce trustworthy guideline recommendations, recommendations that are supported by publication in a top tier journal. The *BMJ* Rapid Recommendations project has recently demonstrated the feasibility and utility of this approach. Here, we are proposing trustworthy nutritional guideline recommendations based on internationally accepted guideline development standards, recommendations that will be informed by rigorous and novel systematic reviews of the benefits and harms associated with nutritional exposures, as well as studies on the values and preferences related to dietary behaviors among members of the international community.

**Discussion:**

Adhering to international guideline standards, conducting high quality systematic reviews, and actively assessing the values and preferences of key stakeholders is expected to improve the quality of nutritional guidelines and their relevance to end-users, particularly patients and community members. We will send our work for peer review, and if found acceptable, we will publish our nutritional recommendations in top-tier general medicine journals.

## Background

### Burden of disease and evidence-based care

Globally, chronic non-communicable illnesses represent the largest burden of disease, and healthy eating habits may be a cornerstone to the prevention and management of chronic disease [1, 2]. Evidence suggests that risk factors related to nutrition have a major impact on disability adjusted life years (DALYs) and death. In the US, estimates suggest that 14% of DALYs and 26% of deaths are attributable to dietary risk factors [3]. Of the 20 leading risk factors for disability in 2010, 13 were directly or indirectly related to diet, including high blood pressure, body-mass index, fasting glucose, and low consumption of fruits and vegetables [4]. The greatest relative burden from chronic diseases such as diabetes, heart disease and cancer may result from or may be exacerbated by poor nutrition [5].

Despite the evidence that nutrition may play a major role in curbing the burden of chronic disease, clinicians typically do not emphasize nutrition in their interactions with patients [6]. The knowledge and time required to apply nutritional counseling in clinical practice comes with a unique set of challenges, including a lack of sufficient nutrition training in medical school and residency programs, and a dearth of both time and financial compensation for offering counseling [7]. Intense media coverage on what is often times low quality, non-systematic collection of evidence (e.g. ecological observations, small clinical studies based on biomarkers) in the field of nutrition serves to compound the problem with a constant source of conflicting messages [8].

Clinicians, including registered dietitians, require primary research and systematic evidence synthesis, including meta-analyses, to understand the available evidence; nutrition practice guidelines to provide appropriate interpretation of evidence and direction in its application; and user-friendly presentations and access to facilitate efficient uptake. Systematic syntheses of evidence are often, however, not available, or the syntheses that do exist are of limited rigor. For example, in systematic reviews and nutrition practice guidelines, important health outcomes (i.e. end points most important to patients and community members, such as mortality, quality of life or dietary satisfaction) are often not optimally identified and synthesized, thus limiting our understanding of the impact of interventions on patient and community members’ lives [9].

Evidence-based nutrition involves the integration of the best available evidence summaries, clinical and public health practice experience, and patient and community values and preferences [10, 11] (Johnston BC et al. The Philosophy of Evidence-Based Principles and Practice in Nutrition. Mayo Clinic Proceedings (submitted August 2018). Within this framework, clinicians can provide optimal care and, when appropriate and possible, engage in shared decision-making with patients, families and members of the community to help them prevent, resolve or cope with their physical, mental, and social health problems.

### The need for trustworthy nutrition guidelines

International groups, including the Institute of Medicine (IoM), Appraisal of Guidelines Research and Evaluation (AGREE), the Grading Recommendations, Assessment, Development and Evaluation (GRADE) working group [12–16], and the Guidelines International Network (GIN) have, using consensus methods, developed recognized and accepted guideline standards. All these standards promote establishing evidence foundations based on systematic reviews and subsequently using these systematic reviews to contribute to ratings of the strength of recommendations [12, 15]. Systematic reviews can offer high or low quality evidence and in either case guideline recommendations can be weak or strong. Table 1 summarizes the IoM standards for trustworthy guidelines.

**Table 1:** Institute of Medicine standards for trustworthy guidelines (2011)

Transparency: details on guideline development and funding are explicit and publicly accessible	
Management of conflicts of interest: prior to finalizing guideline, panelists being considered for membership should declare all interests and activities potentially resulting in conflicts, and all conflicts should be minimized	
Guideline group composition is multidisciplinary with methodological expertise and including patient and community involvement	
Use of systematic reviews for guideline questions	
Establishing evidence foundations for and rating strength of recommendations	
Clear articulation of recommendations	
External review by a full spectrum of stakeholders (e.g. scientific and clinical experts, patients and community representatives)	

Despite these widely accepted standards for trustworthy guidelines and vastly improved methods and processes for their production, systematic surveys of over 1100 practice guidelines across a wide range of health topics indicate that guidelines continue to suffer from important limitations [17–19]. An evaluation of 626 guidelines with the AGREE instrument has demonstrated that despite some increase in quality of guidelines over time, quality scores have, over the last two decades, remained moderate to low [19]. Similarly, an evaluation using IoM standards demonstrated continued poor adherence over the last two decades, with major deficiencies particularly in the management of conflicts of interest [18].

In the field of nutrition, previous systematic evaluations show similar methodological limitations [9, 20–22]. Guidelines issued by several organizations, including authoritative governing bodies, suffer from major limitations in their trustworthiness, relevance and usefulness for practice [9, 23]. For example, using the AGREE instrument, the overall quality of guidelines on nutrition in critically ill adults is suboptimal, with only four of nine guidelines being recommended for clinical use [22]. Using AGREE to assess guideline development across 9 guidelines on daily caloric intake from sugar, although each guideline suggested a decrease in the consumption of foods and beverages containing sugars, the guidelines scored poorly on the AGREE criteria, specifically in rigor of development, applicability, and editorial independence including conflicts of interest [9].

Not only are conflicts of interest an issue in nutritional guideline recommendations, but also in systematic reviews and primary studies [24, 25]. Although underpowered, the results of systematic reviews on the association between sugar-sweetened beverages (SSB) and weight gain or obesity appear to be influenced by industry funding. Among 17 identified systematic reviews, for those reviews without any reported financial conflict of interest, 83.3% (10/12) of conclusions were that SSB consumption could be a potential risk factor for weight gain. In contrast, the same percentage of conclusions, 83.3% (5/6) of those reviews disclosing some financial conflicts with the food and beverage industry indicated that the scientific evidence was insufficient to support a positive association between SSB consumption and weight gain [25]. Further, unique to the field of nutrition, conflicts related to committed dietary behaviours due to personal, family, religious, social, or cultural beliefs can impact the interpretation of results [26]. To optimize the trustworthiness of primary studies, systematic reviews and guideline recommendations in the field of nutrition, the importance of efficiently handling financial, intellectual and other conflicts of interest is paramount, more so because the nutrition field is highly polarized, with strong adherents to ideological stances and evidence of the relationship between financial conflict of interest and authors’ conclusions [26–28].

Another problem is that nutrition guidelines often place excessive trust in the results of observational studies, despite their potentially higher risk of bias [29]. For instance, consider the evidence from a systematic review and meta-analysis of cohort studies versus the only RCT of vitamin C, a micronutrient, for preventing cardiovascular disease. The meta-analysis of nine cohort studies including over 290,000 patients reported a 25% (95% CI 7 to 40%) relative risk reduction in coronary heart disease among men consuming supplemental vitamin C [30]. The Physicians’ Health Study II, a large stand alone RCT of 14,641 participants, found no reduced risk [31]. In considering a systematic review and meta-analyses of cohort studies versus RCTs of antioxidants (i.e. beta-carotene, vitamin A, vitamin C, vitamin E, selenium), results of early cohorts studies among people who consumed a diet rich in these micronutrients demonstrated a lower risk for developing cardiovascular disease and cancer [32], whereas a Cochrane systematic review and meta-analysis of 78 RCTs (*n* = 296,707) revealed no evidence to support antioxidant supplements for primary or secondary prevention of diseases [33]. Over 30 examples of inconsistent results between cohort studies and RCTs exist [34, 35] demonstrating that, although observational studies have important roles in identifying issues for subsequent study and providing guidance prior to the conduct of definitive investigation, the sole reliance on observational studies may result in misleading inferences and recommendations. Systematic reviews of the nutritional literature should consider observational studies separately from randomized trials, with the pooled estimates from the meta-analyses independently assessed for certainty (quality) of evidence.

The GRADE working group has produced standards for the evaluation of the certainty of evidence and for moving from evidence to recommendations that are far more detailed, explicit, transparent and carefully developed than prior systems, and are now in use by more than 100 organizations worldwide [16]. There is evidence that guideline panels making nutritional recommendations are often limited in their experience and ability to use GRADE methods [9, 36]. For instance, despite the fact that WHO made strong recommendations to limit the intake of sugar to below 10% of total daily energy intake, the overall quality of evidence to support recommendations was low to very low [9]. Similar findings have been observed in a review of guideline recommendations across a variety of health care disciplines [37]. With exceptions such as acute life-threatening clinical scenarios (e.g. vitamin K for a patient receiving warfarin with an intracranial bleed and an elevated INR), strong recommendations should not be based on low quality of evidence [15].

Another relevant limitation of many nutrition guidelines involves patient or community participation in the development of recommendations, particularly on the selection of outcomes deemed important to these participants. We systematically identified examples of limited quality guidelines in nine public health guidelines related to sugar consumption. As reflected in the domain stakeholder involvement on the AGREE instrument, many guidelines did not describe how they sought the views and preferences of their target population (patients or the general community), and those that did were vague about the process [9]. Further, guideline panels omitted including members of the general community on the panel, an important component of public health recommendations given the recognition of the importance of patient and community oriented research. For instance, the rationale for the varied sugar intake recommendations gave undue weight to intermediate outcomes including nutrient displacement, and weight gain of the order of 1 kg [9], outcomes not likely of substantial importance to most patients and community members.

### Overcoming the limitations of current nutritional recommendations

Typically, the many organizations that produce guidelines are encumbered by institutional constraints and conflicts of interest, resulting in a profusion of outdated guidelines that do not adhere to recognized standards [12, 13, 16]. A potential solution for the limitations of nutrition guidelines outlined above is for an independent group with clinical and nutritional content expertise and skilled in the methodology of systematic reviews and practice guidelines, but unencumbered by institutional constraints and conflicts of interest, to produce trustworthy recommendations. The *BMJ* Rapid Recommendations project, an initiative of MAGIC (Making GRADE the Irresistible Choice), has recently demonstrated the feasibility and utility of this approach and provided us with the inspiration for NutriRECS (Nutritional Recommendations and accessible Evidence summaries Composed of Systematic reviews) [37]. The NutriRECS working group aims to develop trustworthy nutritional recommendations based on internationally accepted methodological standards [12, 13, 16].

## Objectives

NutriRECS will develop trustworthy guideline recommendations in nutrition. To do so we will include the application of novel and rigorous systematic review and guideline methods using the GRADE approach to investigate the relationship between diets, foods, nutrients and health outcomes; integrate patient and community values and preferences to inform guideline recommendations; apply strict safeguards against conflicts of interest; and use Evidence to Decision (EtD) frameworks to help people use the evidence in a structured and transparent way.

Given the extensive number of research questions and interventions in the broad field of nutrition, NutriRECS will only have the capacity to focus on a small number of guideline projects. Selected projects will be of broad interest to the general public and previous guideline recommendations produced by multiple authoritative organizations will have evidence of extensive methodological limitations (e.g. red and processed meat).

## Methods

### GRADE approach

The GRADE (Grading Recommendations, Assessment, Development and Evaluation) approach is a system for rating the quality of a body of evidence in systematic reviews and grading practice guideline recommendations in health care. GRADE offers a transparent and structured process for developing and presenting systematic reviews, and for carrying out the steps involved in developing recommendations (strong or weak) based on these evidence reviews [16]. Although the certainty of evidence represents a continuum, the GRADE approach results in an assessment of the certainty of a body of evidence into one of four categories (Table 2).

**Table 2:** Certainty of evidence

*GRADE*	*Definition*
*High*	We are very confident that the true effect lies close to that of the estimate of the effect
*Moderate*	We are moderately confident in the effect estimate: The true effect is likely to be close to the estimate of the effect, but there is a possibility that it is substantially different
*Low*	Our confidence in the effect estimate is limited: The true effect may be substantially different from the estimate of the effect
*Very Low*	We have very little confidence in the effect estimate: The true effect is likely to be substantially different from the estimate of effect

Although certainty of evidence is a continuum; GRADE’s discrete categorisation involves some degree of arbitrariness. Nevertheless, advantages of simplicity, transparency, and vividness outweigh these limitations

The strength of a recommendation (strong versus weak) reflects the extent to which a guideline panel is confident that adhering to a recommendation will have more desirable than undesirable consequences, or vice versa, across the range of patients, or members of the public, for whom the recommendation is intended (Table 3). The resulting recommendations would for instance either recommend in favor, or against, people reducing their intake of a particular food or dietary pattern, or, in choosing their diets, not base their choice of foods on these considerations. If appropriate, guideline panels may formulate recommendations on specific subgroups of interest (e.g. those with and without risk factors).

**Table 3:** Implications of strong and weak recommendations for different end-users

*Implications*	*Strong Recommendation*	*Weak Recommendation*
*For patients*	Most individuals in this situation would want the recommended course of action and only a small proportion would not.	The majority of individuals in this situation would want the suggested course of action, but many would not.
*For clinicians*	Most individuals should receive the recommended course of action. Adherence to this recommendation according to the guideline could be used as a quality criterion or performance indicator. Formal decision aids are not likely to be needed to help individuals make decisions consistent with their values and preferences.	Recognize that different choices will be appropriate for different patients, and that you must help each patient arrive at a management decision consistent with her or his values and preferences. Decision aids may well be useful helping individuals making decisions consistent with their values and preferences. Clinicians should expect to spend more time with patients when working towards a decision.
*For policy-makers*	The recommendation can be used to develop policy (e.g. tax on products high in sugar or salt)	Policy-making will require substantial debates and involvement of many stakeholders. Policies are also more likely to vary between regions. Performance indicators would have to focus on the fact that adequate deliberation about the management options has taken place.

### Panel composition and conflict of interest

For each NutriRECS project, panel members will be diverse (i.e. patients and community members, nutritional epidemiologists, research methodologists, primary care physicians, registered dietitians). As the emphasis of NutriRECS will be on producing unbiased recommendations, it will be important to limit conflicts of interest given that conflicts may be associated with conclusions in systematic reviews of nutrition [25, 26], and with nutritional recommendations [9, 21].

Neither the chair nor the methods editor for each NutriRECS guideline project will have any financial conflicts of interest. A financial conflict of interest would include the panel member or a family member of the panel member having received funds directly from a company that produces or promotes a particular dietary pattern, food, or nutrient. NutriRECS members will also be screened for intellectual conflicts that include having authored, co-authored, or held grant funding related to the topic of the guidelines, or have previously expressed strongly held beliefs about the guideline topics. We will also screen potential NutriRECS members for strong personal or religious beliefs related to the guideline remit. Each panel member will submit a full conflict of interest form, which will include declarations of financial, intellectual and other potential conflicts.

We will also closely manage the conflicts of interests among members of the systematic review team and the guideline panel team (no member can have financial conflicts of interest and no more than one-fourth of members can have intellectual conflicts). Up to one third of authors on the systematic review team can participate on the guideline panel to help ensure that panellists are fully informed on the results and nuances of the systematic review. The methods editor will manage interactions between systematic review team and the NutriRECS panel, and submit content to a journal.

### Initial NutriRECS research question and evidence of effects based on systematic reviews

The initial target question for the systematic review to inform the first NutriRECS project on red and processed meat is as follows:

*Among adults, what is the impact of dietary patterns higher in red and processed meat* versus *diets lower in red and processed meat intake (replacement with fish, white meats or vegetarian or vegan diet) on the risk of outcomes important to patients and community members (*i.e. *overall and cardiovascular mortality, cancer, weight, quality of life, satisfaction with diet, type II diabetes, and cardiovascular outcomes [fatal and non-fatal coronary heart disease, non-fatal stroke, non-fatal myocardial infarction, major adverse cardiac events (MACE)]) and on factors that may have a causal relation to cardiovascular outcomes (hypertension and cholesterol), or other adverse outcomes (haemoglobin)?*

With respect to red and processd meat consumption, most dietary guidelines suggest limited consumption of meat because of the reported association with cancer [38–40]. There is, however, a discrepancy between RCTs and observational studies on the topic. Although observational studies tend to show a significant association between red meat consumption and cancer [41], the Women’s Health Initiative, one of the largest RCTs conducted assessing dietary patterns, reported that women consuming a low fat diet reduced red meat consumption by 20% compared to controls, yet there was no effect on multiple cancer types, including colorectal cancer [42, 43]. Despite this, the WHO has indicated that consumption of red meat is “probably carcinogenic” to humans while processed meat is considered carcinogenic to humans [44].

Using the GRADE approach we will begin by working with our NutriRECS panelists to structure and refine our health care questions in terms of the population of interest, and the alternative dietary strategies (e.g. restricted versus unrestricted dietary patterns) based on the most important outcomes to the target audience. For all NutriRECS projects, the target outcomes will be selected based on importance to patients and members of the general community, and will be elicited prior to our conducting the systematic reviews of the literature based on discussions with our NutriRECS panel. Each NutriRECS project panel will have patients and members of the general community to ensure selected outcomes are of importance. Unlike dietary guidelines we are aware of, we will also work with NutriRECS panel to identify subgroups of interest (e.g. age, gender, co-morbidities) and will register the research question and systematic review protocol, including the subgroups of interest, in PROSPERO (https://www.crd.york.ac.uk/prospero/).

In consultation with an expert librarian, we will conduct a systematic search to identify all relevant studies. We will use data from all eligible studies to generate independent estimates of the effect from observational studies and randomized trials, including 95% confidence interval, for all patient or public important outcomes. We will ensure the rigor of the review process by following Cochrane Handbook guidance, including conducting duplicate screening of articles, documenting a priori hypotheses to explain heterogeneity, and conducting formal assessment of risk of bias in duplicate. Further, systematic reviews and meta-analyses of observational studies of food or nutrient intakes (e.g. processed meat), typically presented by generating relative or absolute differences between high consumers (e.g. quantile 5) and low consumers (e.g. quantile 1) of a target exposure will be summarized and evaluated seperately from observational studies that assess dietary patterns (i.e. patterns that are higher in processed meat, and other patterns of food consumption). Subsequently, as a novel method, we will use the summary estimates for each to further asssess etiologic causal inferences. To do so, among outcomes with a statistically significant effect for a food or nutrient, if there is also a significant effect (with similar estimates) based on the dietary pattern data, this will be seen as evidence that undermines the casual inference for the target food or nutrient (e.g. processed meat).

After consultation with the NutriRECS panel members, our research question, subgroups of interest and systematic review methods for red and processed meat and health outcomes has been publically registered with PROSPERO (ID=CRD42017074074).

### Certainty in body of evidence

Subsequently, in duplicate, we will use GRADE methods to rate the certainty of evidence for each outcome across all eligible studies according to study design (randomized trials or observational studies) and five factors that can reduce certainty of evidence (risk of bias, inconsistency of the results, indirectness of the evidence, imprecision and publication bias) and three that can increase certainty (large effect, dose-response gradient, and direction of plausible confounders). The certainty of evidence will reflect our confidence in the estimate of the effect.

Our certainty in the potential desirable and undesirable effects of the interventions based on the body of evidence will then be considered in making a recommendation.

### Values and preferences of patients and community members related to diet restrictions and patterns

Optimal nutritional guideline development requires consideration of patient and community values and preferences associated with dietary patterns. By values and preferences we refer to individuals’ predisposition to either favour (like) or not favour (dislike) something such as red meat [45]. Modifying dietary intake may be accompanied by either despondency or pleasure that affects satisfaction or quality of life. The difficulty many individuals have modifying their diet attests to their attachment to particular dietary practices. To inform NutriRECS work based on values and preferences of patients and community members, we will use the following methods: i) systematic reviews of the literature addressing values and preferences related to diet, and ii) inclusion of patient and public participants on the NutriRECS panel.

We will start by conducting a systematic review of the literature for evidence regarding peoples’ values and preferences in regards to red meat.

### Moving from evidence to recommendations

For moving from the evidence to recommendations we will use the Evidence to Decision (EtD) frameworks. The purpose of Evidence to Decision (EtD) frameworks is to help people use evidence in a structured and transparent way to inform decisions in the context of clinical and public health recommendations. These frameworks summarize evidence, and its certainty, for benefits, harms and burdens, values and preferences, cost considerations, as well as equity, acceptability, and feasibility (Table 4). This approach will ensure that all relevant decisions are transparent to target audiences, thus enabling decision makers in other jurisdictions to adopt or adapt our recommendations [46, 47].

**Table 4:** Evidence to decision framework

	Judgement	Research evidence	Additional considerations
PROBLEM	Is the problem a priority? ○ No ○ Probably no ○ Probably yes ○ Yes ○ Varies ○ Don’t know		
DESIRABLE EFFECTS	How substantial are the desirable anticipated effects? ○ Trivial ○ Small ○ Moderate ○ Large ○ Varies ○ Don’t know		
UNDESIRABLE EFFECTS	How substantial are the undesirable anticipated effects? ○ Large ○ Moderate ○ Small ○ Trivial ○ Varies ○ Don’t know		
CERTAINTY OF EVIDENCE	What is the overall certainty of the evidence of effects? ○ Very low ○ Low ○ Moderate ○ High ○ No included studies		
VALUES	Is there important uncertainty about or variability in how much people value the main outcomes? ○ Important uncertainty or variability ○ Possibly important uncertainty or variability ○ Probably no important uncertainty or variability ○ No important uncertainty or variability		
BALANCE OF EFFECTS	Does the balance between desirable and undesirable effects favor the intervention or the comparison? ○ Favors the comparison ○ Probably favors the comparison ○ Does not favor either the intervention or the comparison ○ Probably favors the intervention ○ Favors the intervention ○ Varies ○ Don’t know		
RESOURCES REQUIRED	How large are the resource requirements (costs)? ○ Large costs ○ Moderate costs ○ Negligible costs and savings ○ Moderate savings ○ Large savings ○ Varies ○ Don’t know		
RESOURCES REQUIRED	What is the certainty of the evidence of resource requirements (costs)? ○ Very low ○ Low ○ Moderate ○ High ○ No included studies		
COST EFFECTIVENESS	Does the cost-effectiveness of the intervention favor the intervention or the comparison? ○ Favors the comparison ○ Probably favors the comparison ○ Does not favor either the intervention or the comparison ○ Probably favors the intervention ○ Favors the intervention ○ Varies ○ No included studies		
EQUITY	What would be the impact on health equity? ○ Reduced ○ Probably reduced ○ Probably no impact ○ Probably increased ○ Increased ○ Varies ○ Don’t know		
ACCEPTABILITY	Is the intervention acceptable to key stakeholders? ○ No ○ Probably no ○ Probably yes ○ Yes ○ Varies ○ Don’t know		
FEASIBILITY	Is the intervention feasible to implement? ○ No ○ Probably no ○ Probably yes ○ Yes ○ Varies ○ Don’t know		

The EtD frameworks have a common structure that includes formulation of the question, an assessment of the evidence for each criterion, and drawing conclusions. EtD frameworks make explicit the criteria that are used to assess interventions or options, the judgments made by each panel member for each criterion, and the research evidence and additional considerations used to inform each judgment. Research evidence refers to facts (actual or asserted) used to inform the panel’s judgments that are derived from studies that used systematic and explicit methods. Additional considerations include other evidence, such as routinely collected data, assumptions, and logic used to make a judgment. As a novel innovation in the field of nutrition guideline development, using EtD frameworks we will survey and collect the anonymous judgements of each panel member prior to making the recommendations and use these judgements for the purpose of panel discussions prior to making the final recommendations. This way, panel members who may be afraid to voice dissenting views during panel meetings will have an opportunity to make their views known. For instance, panel members may make different judgments for one or more subgroups (such as patients who are older or who have more severe disease) in relation to some or all of the criteria.

Afterwards, panels review the judgments they have made for all of the criteria in their assessment and consider the implications of those judgments for the recommendation or decision. Based on their assessment, the panel draws conclusions about the strength of recommendation or type of decision; for example, a strong or weak (sometimes called conditional, discretionary, or qualified) recommendation for or against an intervention or option.

Finally, the panel states the recommendation or decision in a concise, clear and actionable manner, and provides the justification for their recommendation or decision. The conclusions also include relevant considerations about subgroups, implementation, monitoring and evaluation, and research priorities.

### NutriRECS oversight and group process

We have formed an international NutriRECS Executive made up of systematic review and nutrition/public health experts (Dr. Bradley Johnston, Department of Community Health and Epidemiology, Dalhousie University, Halifax, Canada; Dr. Malgorzata M. Bala, Chair of Epidemiology and Preventive Medicine, Department of Hygiene and Dietetics, Jagiellonian University Medical College, Cracow, Poland; Cochrane Poland), experts in guideline methods (Dr. Pablo Alonso-Coello, Iberoamerican Cochrane Centre, Barcelona, Spain; Dr. Gordon Guyatt, McMaster University, Hamilton, Canada), and a community member with guideline experience (Ms. Catherine Marshall, Cochrane Community Representative, New Zealand). The Executive will determine the nutrition topics, the systematic review and practice guideline methods, review and approve conflicts of interest statements and decide whether they are acceptable and, if acceptable, how they will be managed.

Each NutriRECS question will be addressed by a panel including a chair, a methods editor, and upwards of 15 additional panel members from around the world. The chair will be responsible for the management of the NutriRECS guideline panel meetings, while the methods editor will be responsible for assembling the panel and review team, summarizing conflicts of interest for final assessment by the NutriRECS Executive, and creating relevant content (e.g. research questions, subgroups of interest, summary of findings tables based on systematic reviews, surveys using EtD frameworks, and recommendations contextualized based on panelists values and preferences). To produce the systematic reviews, Evidence Profiles, Evidence to Decision frameworks, and user-friendly multi-layered presentation formats (interactive Summary of Findings tables, decision aids), we will use relevant software that has been developed and user tested via randomized trials, surveys and consensus processes [46–50]. Prior to the release of our recommendations we will post the recommendations on our website (www.nutrirecs.com) and seek feedback from members of the public.

### Dissemination plan

Nutritional guidelines and the supporting systematic reviews will be widely disseminated via publication in a high-impact general medicine journal. As well, using GRADE summary of findings tables and decision aids, we will produce user-friendly outputs for clinicians, patients and the community members, including plain language summaries in multiple languages (e.g. Arabic, Cantonese, French, Hindi, Mandarin, Polish, Portuguese, Spanish), and work to ensure these outputs are open-access.

## Discussion

### Main objectives

NutriRECS and corresponding systematic reviews will bring together patients and community members as well as international experts in nutrition and evidence synthesis and translation. Patients, community members and research experts will have the opportunity to reflect on the values and preferences of the communities for which the recommendations are intended in the context of the summary evidence and arrive at nutritional guideline recommendations. Subsequently the guidelines will be sent for peer-review, and recommendations will be publically available via open-access publication.

### Strengths and limitations

The strength of our proposed methods includes our commitment to internationally accepted guideline standards from IoM, GRADE, AGREE and GIN. Our core NutriRECS group includes leading members of these organizations, including GRADE and AGREE. Adherence to these standards will ensure that our recommendations are based on high quality, novel systematic reviews of the literature. Our nutritional recommendations will be put forward by a group of community representatives and experts in nutrition and epidemiology from around the world, representatives and experts with limited to no intellectual or financial conflicts of interest.

Potential limitations of our NutriRECS work include the current lack of resources to ensure that our recommendations remain fully accessible around the world to all potential end-users, including patients and members of the general community, via multiple platforms, and that our systematic reviews and nutritional recommendations remain updated. We are currently seeking funds to regularly update our NutriRECS work, and to have full access to the MAGIC authoring and publication platform (www.magicapp.org) which, based on GRADE evidence summaries, provide decision aids to clinicians, patients and the community on all electronic devices (e.g. laptops, handheld devices). Should we be successful with our funding applications, we will link the scientific journal articles directly to the content in MAGICapp.

A second limitation is sustained funding for our work. NutriRECS is made up of a growing international network of investigators and trainees based in four centres located in Canada (Dalhousie University and McMaster University), Spain (Iberoamerican Cochrane Centre) and Poland (Cochrane Poland). Among the four centres, we have access to an extensive number of local, national, multi-national grant funding opportunities (e.g. Canadian Institutes of Health Research, Heart and Stoke Foundation, Beatrice Hunter Cancer Research Institute, World Cancer Research Foundation). As with almost all independent research programs, we do not have permanent funding for the NutriRECS program. To secure ongoing funding, our team of investigators and trainees consistently write operational grants, career grants and trainee grants. We also sustain ourselves through a large network of research volunteers in exchange for methods training and publication.

A third limitation of our effort is that we have not yet been in contact with organizations producing nutrition guidelines to try and forge a collaboration. Our strategy is to show what can be accomplished in producing trustworthy guidelines by an independent group with limited resources. Having achieved that goal, we believe that this will place us in a more credible position when interacting with established organizations.

## Conclusions

The implications of the NutriRECS project include the promotion of better-informed decision-making by patients, members of the community, clinicians, and public health policy-makers on the desirable and undesirable effects of alternative dietary patterns, as well as foods and nutrients on important health outcomes.

## Abbreviations

AGREE: Appraisal of Guidelines Research and Evaluation
EtD: Evidence to Decision
GIN: Guidelines International Network
GRADE: Grading Recommendations, Assessment, Development and Evaluation
INR: International Normalized Ratio
IoM: Institute of Medicine
NutriRECS: Nutritional Recommendations and accessible Evidence summaries Composed of Systematic reviews
