# Correction to: Methods for trustworthy nutritional recommendations NutriRECS (Nutritional Recommendations and accessible Evidence summaries Composed of Systematic reviews): a protocol

**DOI:** 10.1186/s12874-019-0888-4

**Published:** 2019-12-17

**Authors:** Bradley C. Johnston, Pablo Alonso-Coello, Malgorzata M. Bala, Dena Zeraatkar, Montserrat Rabassa, Claudia Valli, Catherine Marshall, Regina El Dib, Robin W. M. Vernooij, Per O. Vandvik, Gordon H. Guyatt

**Affiliations:** 1 Department of Community Health and Epidemiology, Faculty of Medicine, Dalhousie University, Halifax, Canada; 2 Department of Health Research Methods, Evidence and Impact, McMaster University, Hamilton, ON Canada; 3 Iberoamerican Cochrane Centre Barcelona, Biomedical Research Institute San Pau (IIB Sant Pau), Barcelona, Spain; 4 CIBER de Epidemiología y Salud Pública (CIBERESP), Barcelona, Spain; 5 Chair of Epidemiology and Preventive Medicine, Department of Hygiene and Dietetics, Jagiellonian University Medical College, Krakow, Poland; 6 Cochrane Consumer and Honorary Patron of the Guidelines International Network, Wellington, New Zealand; 7 Institute of Science and Technology, Unesp - Univ Estadual Paulista, São José dos Campos, Brazil; 8 Department of Research, Netherlands Comprehensive Cancer Organisation (IKNL), Utrecht, The Netherlands; 9 Institute of Health and Society, Faculty of Medicine, University of Oslo, Oslo, Norway; 10 Department of Medicine, Innlandet Hospital Trust-division, Gjøvik, Norway; 11 Department of Medicine, McMaster University, Hamilton, ON Canada

**Correction to: BMC Med Res Methodol (2018) 18:162**

**https://doi.org/10.1186/s12874-018-0621-8**

Following publication of the original article [1], the authors reported a change in the ‘Competing interests’ section as described below.

‘Competing interests’ in the original article:

- BCJ, PAC, POV and GG are members of the GRADE working group. Remaining authors declare no competing interests.

Revised ‘Competing interests’:

- BCJ, PAC, POV and GG are members of the GRADE working group. In 2015, BCJ received funding from the International Life Science Institute to assess the methodological quality of nutrition guidelines using internationally accepted GRADE and AGREE guideline standards for a study titled “The Scientific Basis of Guideline Recommendations on Sugar Intake: A Systematic Review” [2]. The remaining authors declare no potential competing interests.
